# Supplementary figures and images for: Genome-Wide Identification and Analysis of the MADS-Box Gene Family in Theobroma cacao
Source: Genes (Basel). 2021 Nov 15;12(11):1799. doi: 10.3390/genes12111799 (PMC8622960; doi:10.3390/genes12111799)

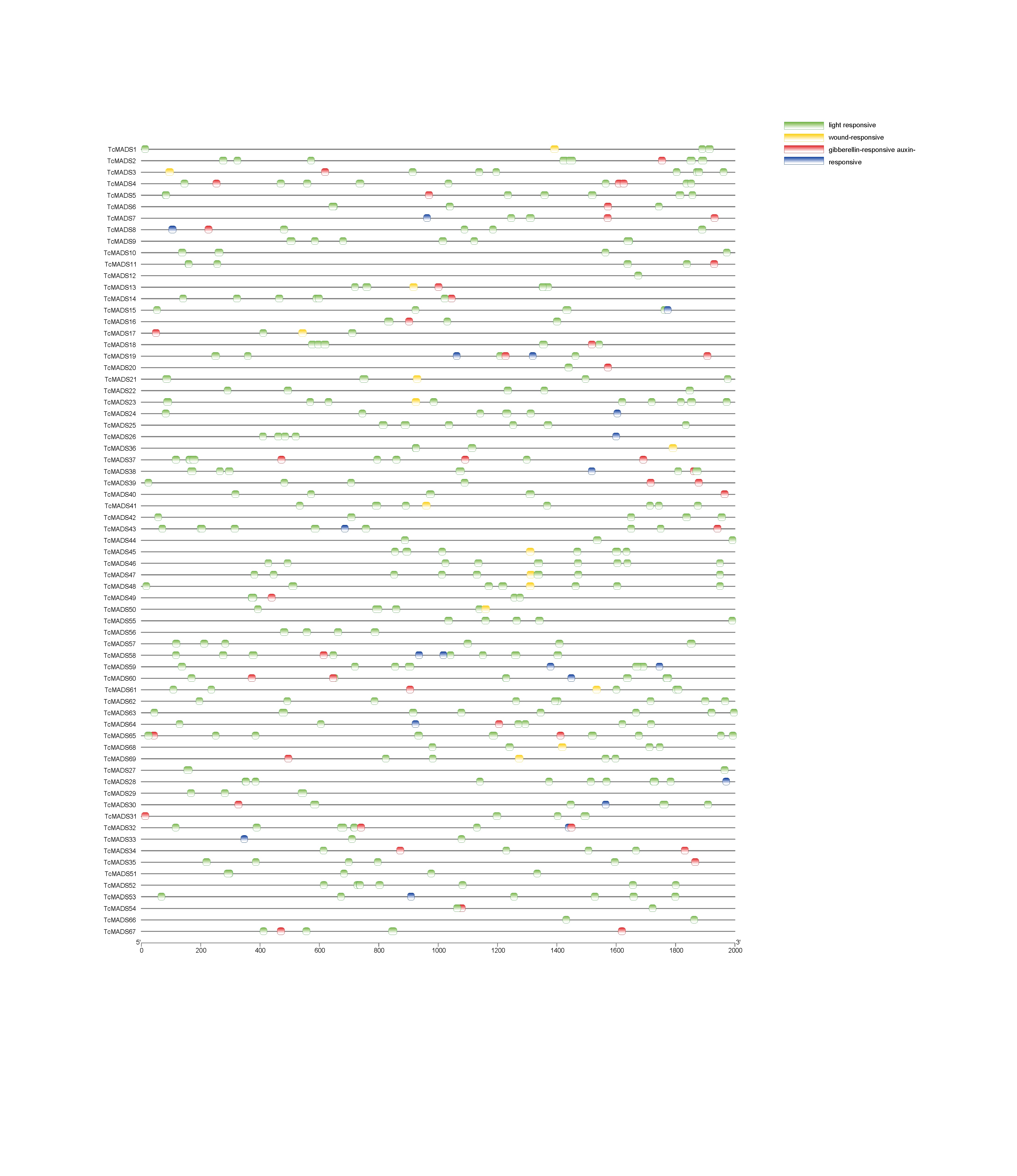

Supplement: Supplementary file 1 [file genes-12-01799-s001.zip › genes-1439884-supplementary/Supplementary Files/Supplementary File3-Predicted cis-elements in MADS-boxs promoters.jpg]
